# Impact of mobile radiography services in nursing homes on the utilisation of diagnostic imaging procedures

**DOI:** 10.1186/s12913-019-4276-x

**Published:** 2019-06-26

**Authors:** Elin Kjelle, Kristin Bakke Lysdahl, Hilde Merete Olerud

**Affiliations:** Faculty of Health and Social Sciences, University of South-Eastern Norway, Pb 235, 3603 Kongsberg, Norway

**Keywords:** Mobile radiography service, Nursing home, Nursing home resident, Telemedicine, Diagnostic imaging, Mobile health unit

## Abstract

**Background:**

In the last decade, mobile radiography services have been introduced in nursing homes in several countries. Earlier research found an underutilisation of diagnostic imaging among nursing home residents. However, the effects of introducing mobile radiography services on the use of diagnostic imaging are unknown. The purpose of this study was to determine the utilisation of diagnostic imaging among nursing home residents and if there are any differences between hospitals with and without a mobile radiography service.

**Methods:**

Data for 2015 were collected from the radiological information systems of 11 hospitals. The data included information on the anatomical region/organ/organ system, modality, and information on where the examination took place. Using nursing home beds as a proxy for nursing home residents’ differences in the use of diagnostic imaging in areas with hospitals with and without mobile radiography services were analysed. The chi-squared test was used to compare the areas.

**Results:**

From 11,066 examinations of nursing home residents, 87% were plain radiographs, 8% were CT scans, and 4% were ultrasound examinations. In areas with mobile radiography services, there was a significantly higher proportion of diagnostic imaging used per nursing home bed, 50% per bed compared to 36% per bed in areas without; *p =* < 0.001. Furthermore, in areas with mobile radiography services, there was a significantly lower proportion of CT and ultrasound used per nursing home bed, 2.5 and 1.4% respectively per bed compared to 4.7 and 2.2% respectively per bed in areas without; *p =* < 0.001.

**Conclusions:**

This study demonstrate a lower use of radiology by nursing home residents compared to the general population, and indicates that mobile radiography services increase the level closer to the user rate in the general population. The proportions of plain radiographs are significantly higher in areas with a mobile radiography service, while the proportion of more advanced imaging techniques such as CT and ultrasound are lower. The higher use of diagnostic imaging is most likely appropriate because of higher morbidity and lower use of diagnostic imaging among nursing home residents, compared to the general population. Further research is necessary on how to improve diagnostic imaging services for nursing home residents.

## Background

According to the European Union demography report [1] an increase in the old age population is expected over the few next decades, which may lead to an increase in people living in healthcare institutions [1, 2]. Demographic changes in the Western world makes high-quality and cost-effective services an important factor in order to maintain sustainable healthcare systems [3, 4]. In 2015, 42,644 people resided in care institutions for the elderly in Norway, including both long- and short-term stays. Residents in different types of care institutions in Norway, hereafter called nursing homes, have a mean age of 82.3 years [5]. These residents have a greater need for specialised healthcare services compared to the rest of the population. This is due to the fact that up to 80% are living with dementia and/or several co-morbidities and high incidence of acute illness and injuries [6–11]. According to Graverholt et al. [8], 16–62% of nursing home residents are admitted to a hospital for acute care every year, of which up to 40% could be avoided. Admittance to hospital is associated with complications for nursing homes residents, thus treatment in the nursing home is preferable [6, 7]. Nursing home residents can be admitted to a hospital for three reasons: diagnostic, treatment to improve function and life expectancy, or palliative treatment [6]. In 2013, the most common reasons for acute admittance of nursing home residents in a Norwegian setting were diseases of the respiratory system (19.8%), injury, poisoning, and certain other consequences of external causes (mostly fall injuries and hip fractures) (17.8%), diseases of the circulatory system (16.5%), and diseases of the digestive system (9.9%) [8]. According to Ranhoff and Linnsund [6], there are two cases where hospitalisation benefits most nursing home residents: hip fractures and severe anaemia. Otherwise, the benefit of admittance depends on the residents’ condition and most would be better off treated in the nursing home [6]. According to Wang et al. [12], approximately 72% of nursing home residents visiting an emergency department in the USA needed diagnostic imaging; of these, approximately 85% needed X-ray examinations and 35% needed CT scans. In Oslo in 2004 during a period of 8 weeks 51% of health incidents in nursing homes included diagnostic imaging, a proportion of about 0.5 examination per person per year [13]. 90% of these examinations were plain radiography, 4% were CT of the head, 4% fluoroscopy and 2% ultrasound [13]. Diagnostic imaging provide evidence that adds to treatment or care of nursing home residents. Most examinations of nursing home residents are performed in question of fractures and increased dyspnoea [11, 14, 15]. According to several studies from Norway and Sweden 29–85% of diagnostic imaging procedures affected the treatment and/or care of the resident, by either confirming or disconfirming the suspected diagnosis, provide evidence for unknown pathology or provide status for follow-up purposes [13–15]. Not having access to diagnostic imaging could thus result in inaccurate treatment, pain, and reduced life quality for these residents [6, 7, 11].

In the general population, international trends in diagnostic imaging show a decrease in the use of plain radiographs and fluoroscopy and an increase in the use of CT and MRI [16–21]. However, for nursing home residents, plain radiography seem to be the most important imaging test [12, 13, 22]. Furthermore, Lærum, Åmdal [11] indicated an underuse of diagnostic imaging for nursing home residents compared to the general population. This is quite the opposite of expectations based on nursing home residents’ health status [11]. The reason for this underuse may be that some residents are in no condition to travel to the imaging department or there is a lack of personnel to accompany residents [13, 14], thus inferior access to imaging. Earlier research showed that access to imaging services influenced the utilisation rate [23, 24].

To improve access, a mobile radiography service to nursing homes was piloted in Oslo in 2004 [11]. In this service, a radiographer brought a portable X-ray machine and conducted plain radiographs (skeletal, chest or abdominal images) in the residents’ rooms [25]. At present, such services operate in Australia, Italy, Norway, Sweden, and Switzerland [22, 25–29]. According to earlier research, mobile radiography is beneficial for nursing home residents with a reduction in onset delirium, fewer hospital admittances and more adequate treatment, further mobile radiography services reduce societal and healthcare costs [14, 25, 30, 31].

At present, the general population receives 0.9 examinations per inhabitant in Norway [32]. However, we do not know how mobile radiography services affect the number and type of diagnostic imaging examinations nursing home residents receive. Information on the utilisation of diagnostic imaging may provide a better basis for designing healthcare services for this fragile patient group. The aim of this study was to describe the overall utilisation of diagnostic imaging in the population of nursing home residents and to explore if there are any differences between the type and number of examinations provided by hospitals with and without mobile radiography services.

## Methods

### Data collection

Data were requested from the radiology information systems (RIS) of 12 different hospitals/hospital trusts from the four healthcare regions of Norway. Six had a mobile radiography service combined with a hospital-based service and six had a hospital-based service only. Eleven hospitals delivered data and are included in the study, six without and five with mobile services in the surrounding areas.

The data included all diagnostic imaging procedures for nursing home residents in 2015. The data were recorded using the Norwegian classification of radiological procedures (NCRP) [33]. This system provides detailed information on anatomical region/organ/organ system, modality, and whether the examination was for diagnostic or treatment purposes [33]. The data were allocated to the following categories: plain radiographs (2D images), computed tomography (CT), ultrasound, magnetic resonance imaging (MRI), nuclear medicine, and others (including fluoroscopy, interventional procedures, and mammography). In addition, information on the place of examination (a hospital or a nursing home) was provided. All of the hospitals except one provided full datasets. This hospital provided data on the mobile service only.

The hospitals and the areas they cover with respect to imaging services were sorted into two categories, those with mobile radiography services and those without. Information on these two categories is presented in Table 1. In the category with mobile radiography service, three of the five mobile services did not cover all of the nursing homes in their area. This differs with each hospital’s organisation of the mobile radiography service and contracts with municipalities [34]. There are twice as many private imaging services in the areas with mobile radiography services [35–38], these were not included in the study. The number of overall types of examination are presented in plain numbers. For the analyses comparing the examination rates between the two categories, aggregated data on the nursing home beds in the areas the hospitals cover was used as the denominator, that is, the calculated proportion of the examinations per bed. Nursing home beds were used as a proxy for the nursing home residents, as the RIS data from the hospitals did not include information on the individual residents. The number of residents may be higher than the number of beds due to short or intermediate time stays or residents dying. There were 24,805 nursing home beds in 605 nursing homes in the areas covered by the included hospitals in 2015 [39, 40]. About 31,600 persons resided in these nursing homes in 2015 and about 28% of these residents had short or intermediate time stays [41].

**Table 1:** Information on the included areas covered by hospitals with and without mobile radiography services

	With mobile radiography service (% covered by mobile service)	Without mobile radiography service
Nursing homes, *n*	310	295
Nursing home beds, *n*	14,500 (86)	10,305
Mean population density (per km^2^)	412.1	125.5
Proportion of short time residents	28.6%	28.9%
Private imaging services, *n*	17	8

### Statistical analysis

To perform the comparative analysis, the data were divided into the two categories, with or without mobile radiography service. Microsoft Excel (2013) was used to assess the descriptive statistics. R (R Core Team, 2018) was used to perform 2-tailed chi-squared tests in the difference of proportion. *P* values less than 0.05 were considered significant.

### Ethics

Regional Committees for Medical and Health Research Ethics (REC) approved the dispensation from professional secrecy (12 February 2016, project no. 2468) and the Norwegian Centre for Research Data (NSD) approved the data collection and handling for this study (15 February 2016, project no. 45571). In addition, each hospital’s local research committee approved the data collection.

## Results

**Fig. 1:**
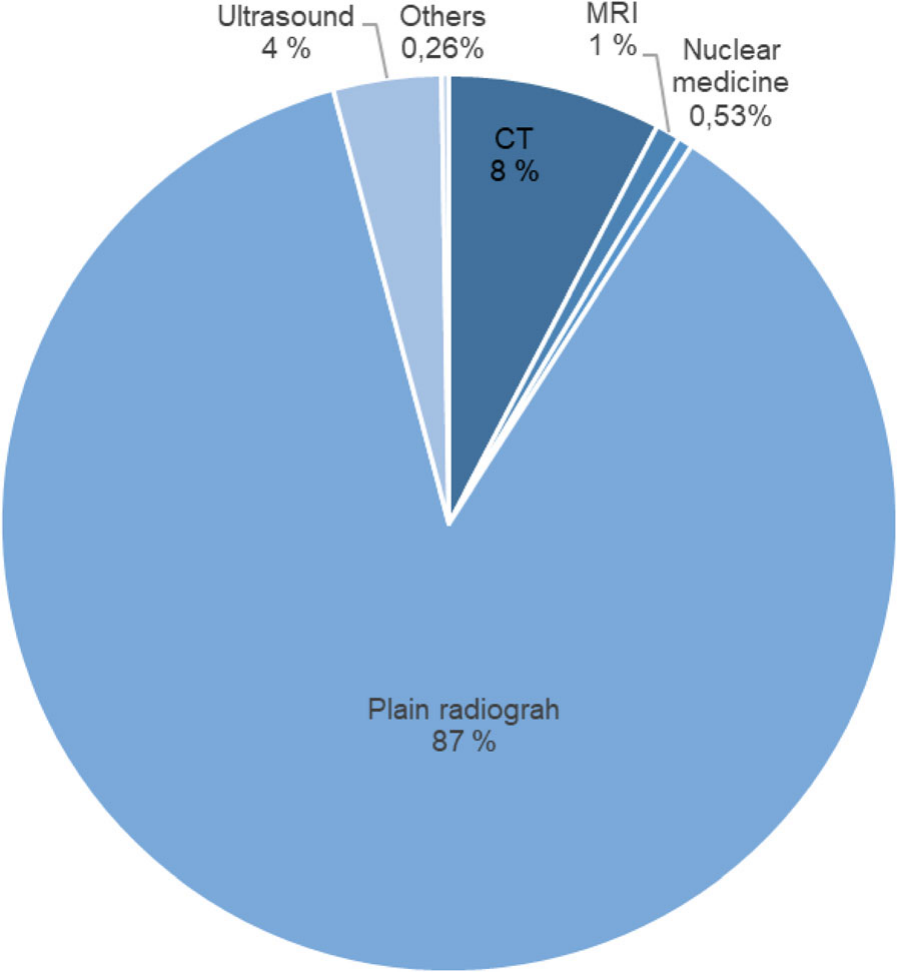
Distribution of the 11,066 examinations performed for nursing home residents according to modality, in 2015

A total of 11,066 diagnostic imaging examinations were performed by the included hospitals on nursing home residents in 2015. The hospitals covered 24,805 nursing home beds, for an average number of 0.45 examinations per nursing home bed. Figure 1 shows the proportion per modality. Plain radiographs were the most common examination (87%), followed by CT (8%) and ultrasound (4%) as the third most common examination. MRI, nuclear medicine, and others (fluoroscopy, interventional procedures, and mammography) accounted for 1% or less of the examinations performed.

### Overall use of diagnostic imaging

There were 14,500 nursing home beds in areas with mobile radiography services and 10,305 nursing home beds in areas without mobile radiography. Using the chi-squared test, the two categories were compared and the results are presented in Table 2. Out of all of the examinations, the proportion was significantly higher in the category with mobile radiography services, 50% per bed compared to the category without, 36% per bed (χ^2^(df = 1, *n* = 11,066) = 470.39, *p =* < 0.001). In other words, up to half of the residents occupying the nursing home beds were likely to receive an examination during 1 year. The proportion of plain radiograph examinations was significantly higher in the category with mobile radiography services, 45.8% compared to 28.7% without (χ^2^ (df = 1, *n* = 9600) = 736.22, *p =* < 0.001). In the category with mobile radiography services, the proportion of CT and ultrasound examinations was significantly lower: 4.7% without mobile services and 2.5% with and 2.2% without and 1.4% with (χ^2^ (df = 1, *n* = 851) = 83.33, *p =* < 0.001 and χ^2^ (df = 1, *n* = 431) = 20.08, *p =* < 0.001), respectively. Nuclear medicine, MRI, and other examinations showed no statistically significant differences in proportion.

**Table 2:** Comparison of the proportion of examinations per nursing home bed in areas with and without mobile radiography services

	With mobile radiography service		Without mobile radiography service		Diff. (% point)	95% Confidence interval		*P* value
	*n*	Proportion of examinations per bed (%)	*n*	Proportion of examinations per bed (%)		Min.	Max.	
Nursing home beds	14,500		10,305					
All examinations	7306	50.00	3760	36.00	14.0	12.7	15.10	< 0.001
Plain radiographs	6638	45.80	2962	28.70	17.10	15.8	18.20	< 0.001
CT	368	2.50	483	4.70	−2.20	−2.6	−1.70	< 0.001
Ultrasound	206	1.40	225	2.20	−0.80	−1.0	−0.40	< 0.001
MRI	53	0.37	43	0.42	−0.05	− 0.2	0.10	0.587
Nuclear medicine	27	0.20	32	0.30	−0.10	− 0.3	0.01	0.065
Other	14	0.10	15	0.15	−0.05	− 0.15	0.05	0.355

### Plain radiographs

**Fig. 2:**
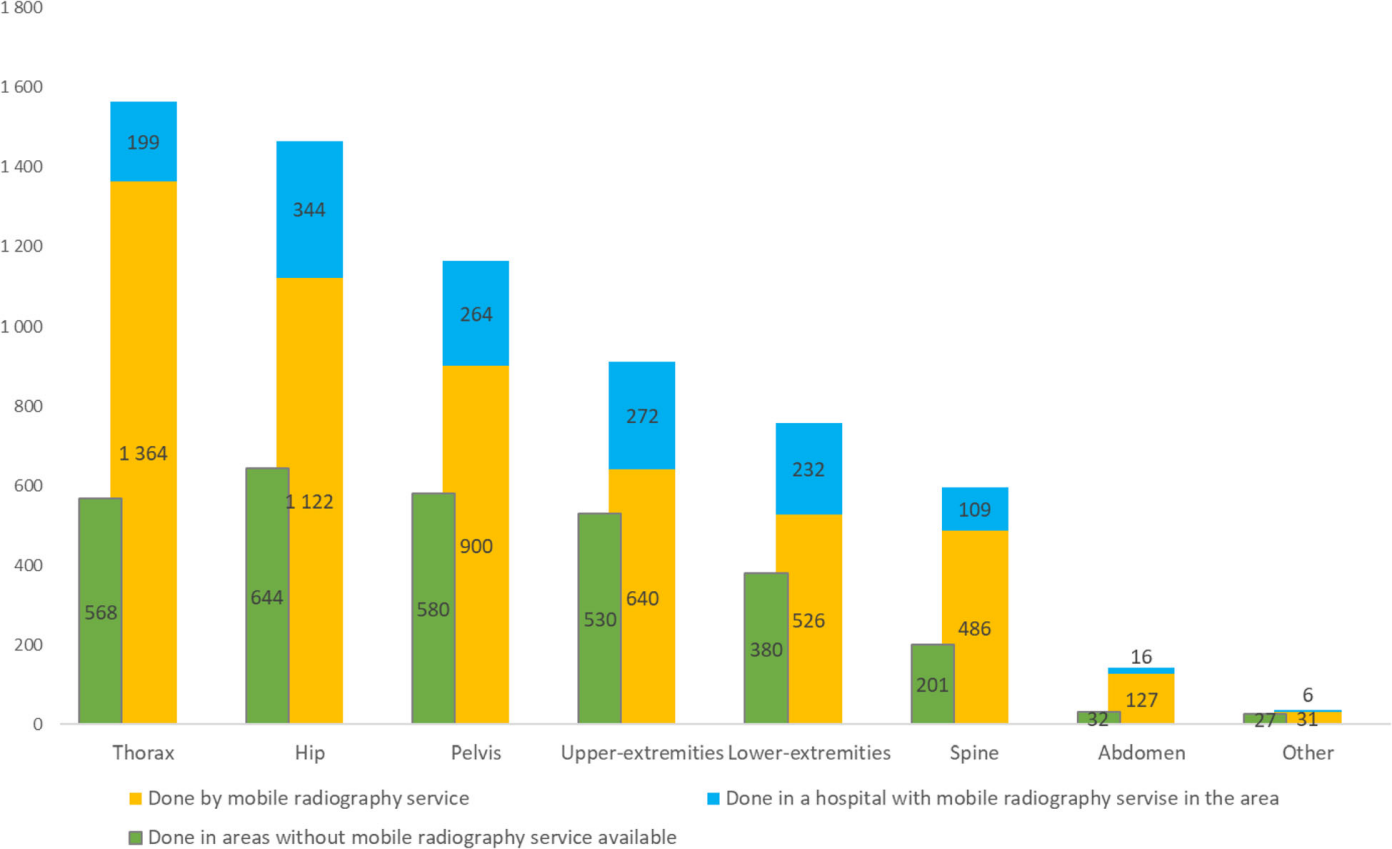
Overview of the types of plain radiograph examinations in areas with and without mobile services. “Other” includes examination types with a frequency of < 20 per year

A total of 9600 plain radiographs were conducted on nursing home residents. The most frequent examinations were the chest, hip, and pelvis, which constituted just over 22% (*n* = 2131), 22% (*n* = 2110), and approximately 18% (*n* = 1744), respectively. These were followed by approximately 27% (*n* = 2580) of examinations of the extremities (for example, the shoulder, wrist, ankle, and foot), spine just over 8% (*n* = 796), and abdomen approximately 2% (*n* = 175). Examinations of for example the sternum, scapula, and clavicular bone with a total score of < 20 examinations per year have been grouped together in “Other”; these amounted to < 1% of the examinations. Figure 2 shows the number of different examinations performed by mobile radiography services and by hospitals in areas with and without mobile services, respectively. Overall 6638 of the examinations were in the category with mobile radiography services, and 78% (*n* = 5196) of the plain radiographs were performed by the mobile radiography service.

In Table 3 the plain radiography examinations in the category with mobile radiograph services is presented per site of examination. The mobile services does all sorts of 2D plain radiography examinations without the use of contrast media. The largest proportion of examinations performed in nursing homes was abdominal examination (88%). Further, 87% of chest examinations and 69–84% of skeletal examinations were performed in nursing homes.

**Table 3:** An overview of types of plain radiography examinations performed in the category with mobile radiography services divided by site of examination

	In hospital	At nursing home	Total	Proportion at nursing home
Chest	199	1364	1563	87%
Hip	344	1122	1466	76%
Pelvis	264	900	1164	77%
Upper-extremities	272	640	912	70%
Lower- extremities	232	526	758	69%
Colum	109	486	595	82%
Other skeletal^a^	6	31	37	84%
Abdominal	16	127	143	88%
Total	1442	5196	6638	78%

^a^Clavicular, sternum, scapula etc

### CT

There were 851 CT examinations performed on nursing home residents in 2015. Approximately 41% (*n* = 354) of the CT examinations were of the brain and approximately 24% (*n* = 203) were of the abdominal/pelvic areas (Table 4). Furthermore, approximately 21% of the examinations were combined CT examinations (*n* = 72), CTs of the spine (*n* = 45), and CTs of the extremities (*n* = 31). Examinations of for example the urinary system and angiograms with a frequency of < 20 examinations per year have been grouped together in “CT other” and constituted just over 9% of the examinations.

**Table 4:** An overview of types of CT examinations performed at hospital divided by category and in total

	With mobile radiography service, *n*	Without mobile radiography service, *n*	Total (%)
CT brain	153	201	354 (41.6)
CT abdomen/pelvis	79	124	203 (23.9)
CT combinations	34	38	72 (8.5)
CT chest	35	33	68 (8.0)
CT spine	22	23	45 (5.3)
CT extremities	9	22	31 (3.5)
CT other^a^	36	42	78 (9.2)
Total	368	483	851 (100)

^a^Examination types with a frequency of < 20 per year

### Ultrasound examinations

Of the 431 ultrasound examinations performed on nursing home residents, approximately 36% were examinations of the veins in the lower extremities (*n* = 156) and approximately 33% were of the abdominal organs (*n* = 143) (Table 5). Furthermore, just over 8% were examinations of the urinary system (*n* = 36) and approximately 7% were of the mammae/axilla (*n* = 32). Examinations of for example the skin and male genitalia with a frequency of < 20 examinations per year have been grouped together in “Ultrasound other” and amounted to just under 15% of the examinations.

**Table 5:** An overview of types of ultrasound examinations at hospital divided by category and in total

	With mobile radiography service, *n*	Without mobile radiography service, *n*	Total (%)
Ultrasound veins in lower extremities	80	76	156 (36.2)
Ultrasound abdominal area	61	82	143 (33.2)
Ultrasound urinary system	12	24	36 (8.3)
Ultrasound mammae/axilla	21	11	32 (7.4)
Ultrasound other^a^	32	32	64 (14.9)
Total	206	225	431 (100.0)

^a^Examination types with a frequency of < 20 per year

## Discussion

### Overall use of diagnostic imaging

Diagnostic imaging benefits nursing home residents by enabling adequate treatment and care, and reduce hospitalisations [13–15]. One would expect nursing home residents living with a higher number of co-morbidities and with an increased rate of acute illness compared to the general population to have a higher rate of diagnostic imaging procedures [8, 13]. In the general population of Norway where an average of 0.9 examinations are performed annually per inhabitant [31]. In this study of the population of nursing homes there were 0.36–0.5 imaging procedures performed per nursing home bed per year. Assuming there are more residents than beds during the cause of 1 year, nursing home residents receive less than half the proportion of examinations. This is in line with earlier research that indicates an underuse of diagnostic imaging in the nursing home population due to their higher need for health services [11].

Earlier research explained the underuse to be caused by the fact that 10–20% of residents were unable to undergo an appropriate examination at a hospital [11, 14, 25]. This was due to either the resident’s condition or a lack of personnel/family to accompany them [11, 25]. Our study demonstrate a higher proportion in the use of diagnostic imaging in general when mobile radiography is available. Earlier research showed that easier access to imaging services increases their use [11, 14, 23, 24]. With a mobile radiography service, nursing home residents would have easier access to imaging services. The higher use of imaging in areas with mobile radiography may therefore be explained by the existence of this service.

About 38% of residents examined by the mobile service need treatment in hospital [14, 15]. In such cases there is the possibility of examinations being repeated if there is an examination performed in nursing home and the resident is later transferred to hospital. This would increase the proportion of examinations in areas with mobile radiography services, probably without benefits to the patient. The repeat rate is not available in this study, however in Eklund et al’s [15] study 1 out of 241 examinations were repeated. This repeat was due to inferior image quality. Earlier research have shown mobile radiography examinations to have similar or adequate image quality compared to hospital imaging [25]. However, mobile equipment have inherent limitations which could lead to inferior image quality on for instance obese patients [42]. More knowledge is needed on these retakes after mobile examinations, however the amount of retakes seems small and would not affect the results of this study.

### Use of modalities and clinical indications

Earlier research indicated that plain radiographs were the most important type of examination for nursing home residents [8, 12]. In this study, 87% of the examinations were plain radiographs. In the areas with mobile radiography services, the proportion of plain radiographs was approximately 46%, while the proportion was almost 29% in areas without this service. Examinations of the chest, hip, pelvis, and extremities were the most common. This is consistent with the conditions for which nursing home residents are most commonly admitted to hospital [8]. The mobile services could do all types of plain radiographs and the proportions done were 78–80% for skeletal while the chest and abdominal images were 87 and 88% respectively. This may be because fractures need to be treated in hospital, while infection could be treated in the nursing homes [6–10].

CT examinations of the head and abdominal/pelvic area were the most common. In ultrasound, the veins of the lower extremities and the abdominal organs were most commonly examined. When a mobile radiography service was available, there was a significantly smaller proportion of CT and ultrasound. This indicated that residents transferred to a hospital are more likely to be examined with more advanced imaging technologies. Again, this complies with earlier research showing that better access increased use [23, 24]. This difference could indicate that residents transferred to a hospital slightly overuse CT and ultrasound examinations beyond what is needed to obtain a diagnosis. On the other hand, there may be an underuse of more advanced imaging when a mobile radiography service is present.

There were no data on the medical indication for the examinations available in this study; thus, the appropriateness could not be assessed. However, earlier research on nursing home residents’ reasons for admittance showed that respiratory infections, chronic obstructive pulmonary disease, injuries (fractures), and diseases of the circulatory system were the most common conditions [6, 8]. According to Ranhoff and Linnsund [6], with most of these conditions except hip fractures, hospitalisation does not benefit most residents as long as adequate treatment is provided in the nursing home. Adequate nursing home treatment also benefits residents on a psychosocial level [25, 43]. Nursing home residents and especially those living with dementia need a familiar environment to feel safe and cared for [43, 44]. Transfer to a hospital for an examination or admission could cause exhaustion, delirium, and/or injuries [7, 9, 11, 28, 44]. Thus, residents may have a higher quality of life when transfer to a hospital can be avoided [43].

To increase access to imaging technologies for nursing home residents, there is the possibility of having mobile CT or ultrasound units. Mobile CT units in ambulances for diagnosing stroke patients are available in Germany, Norway, and the US [45–47]. CT of the head amounts to > 40% of CT examinations of nursing home residents, so using these mobile CT units could help increase access and at the same time reduce unnecessary transfers to a hospital. Ultrasound machines are easier to transfer than both CT and X-ray equipment. With qualified personnel on the mobile radiography service, both plain radiographs and ultrasound examinations could be performed by the same mobile unit, thus further reducing the number of resident transfers to a hospital.

### Limitations of the study

The sample used in this study included examinations of nursing home residents from 11 hospitals. Data from before the implementation of mobile radiography services from these areas, which would have been preferable, were not available. Instead data from areas with and without mobile radiography services was used. Data were collected from hospitals in different parts of Norway, with different population densities and different travel distances from nursing homes to a hospital. Travel distance may affect the use of mobile radiography services as well as the frequency of transfers to a hospital due to the effect of easy access [23, 24]. Mobile radiography services in Norway are established in urban areas [25]. Thus, some of the increased use of diagnostic imaging could be explained by easier access to imaging services [23].

On the other hand, there are several differences in the categories underestimating nursing home residents’ use of diagnostic imaging in areas with mobile radiography services. Firstly, there are twice as many private hospitals and imaging centres from which no data were collected. Secondly, two of the five hospitals with mobile radiography services provided this service to all of the surrounding nursing homes. The other three did not fully cover the surrounding nursing homes [34], amounting to 86% coverage. With full coverage the access would increase further. Thirdly, there is a small difference in the number of nursing homes between the categories, however there are far more nursing homes beds in the category with mobile radiography services. Thus, there are larger nursing homes with more beds per nursing home in this category. The size of nursing home may affect services available, the referral rate, and types of treatments available in the nursing home. Graverholt et al. [8] found that smaller nursing homes have a higher hospital admission rate than larger nursing homes, however how nursing homes size affect referral rate for diagnostic imaging is unknown. Under the assumption that a higher admission rate equals a higher referral rate for diagnostic imaging, the proportion of examinations may be under-estimated in the category with mobile radiography services. However, this may be counteracted by increased access to diagnostic imaging in urban areas [23]. Fourthly and lastly, one hospital presented data on the mobile radiography service only. To obtain a better overview of the area with missing data, the hospital reporting mobile services were combined with data from another hospital in the same city to represent the surrounding area in question, however, the total numbers of examinations were underestimated in this area. Taking into account all these effects of differences between the categories, there is most likely no over estimation of examinations in areas with mobile radiography services.

Notwithstanding the limitations, there is a good basis for comparing the two categories. The different hospitals used the same coding system; this secured a uniform data format and assisted an accurate count. Further, individual physician’s referral rates would fluctuate in both categories and the proportion of short-stay residents are equal in the two categories. This supports the use of nursing home beds as a proxy for residents, excluding the bias of proportion of short-time beds affecting the referral rates, in the same way as affecting hospitalisation rates [8]. In addition, there is about the same number of nursing homes in the categories and all parts of the country were included, rendering the results valid and reliable.

## Conclusions

This study demonstrate a lower frequency of radiology in the group of nursing home residents compared to the general population and indicate that mobile radiography services influence the use of diagnostic imaging. There was a substantial difference in the use of imaging services between the categories. With mobile radiography services, the proportion of imaging used per nursing home bed are higher than without. This was due to a greater proportion of plain radiographs in areas with mobile radiography services. Nursing home residents needed plain radiographs in 87% of the examinations. Chest, hip, pelvis, and the extremities were most common plain radiograph examinations performed. Furthermore, the proportion of CT and ultrasound examinations were significantly lower when a mobile radiography service was available. The findings indicated that mobile radiography services meets a need for increased access to diagnostic imaging for nursing home residents and suggested possibilities for expanding services to include CT and ultrasound. However, further research is necessary on how to improve diagnostic imaging services for nursing home residents.

## Data Availability

The dataset generated and analysed during the current study are not publicly available due to participant anonymity issues, but are available from the corresponding author on reasonable request.

## Abbreviations

CT: Computed Tomography
MRI: Magnetic resonance imaging
RIS: Radiology Information System
